# Immediate and short-term effects of continuous theta burst transcranial magnetic stimulation over contralesional premotor area on post-stroke spasticity in patients with severe hemiplegia: Study protocol for a randomized controlled trial

**DOI:** 10.3389/fneur.2022.895580

**Published:** 2022-08-23

**Authors:** Xiupan Wei, Nan Xia, Yang-An Li, Minghui Gu, Tongming Zhang, Wei Gao, Yali Liu

**Affiliations:** ^1^Department of Rehabilitation Medicine, Tongji Hospital, Tongji Medical College, Huazhong University of Science and Technology, Wuhan, China; ^2^World Health Organization Collaborating Centre for Training and Research in Rehabilitation, Wuhan, China; ^3^Department of Traumatic Surgery, Tongji Hospital, Tongji Medical College, Huazhong University of Science and Technology, Wuhan, China

**Keywords:** spasticity, stroke, premotor area, theta burst stimulation (TBS), H reflex, randomized controlled trial, diffusion tensor imaging (DTI)

## Abstract

**Background:**

Post-stroke spasticity is an important complication that greatly affects survivors' functional prognosis and daily activities. Increasing evidence points to aberrant contralesional neuromodulation compensation after brain injury as a possible culprit for increased spasticity in patients with severe stroke. Hyperactivity of the contralesional premotor area (cPMA) was supposed to be highly correlated with this progression. This study aims to demonstrate the immediate and short-term efficacy of continuous theta-burst stimulation (cTBS) targeting cPMA on upper limb spasticity in severe subacute stroke patients.

**Methods:**

This trial is a single-center, prospective, three-group randomized controlled trial. Forty-five eligible patients will be recruited and randomized into three groups: the sham-cTBS group (sham cTBS targeting contralesional PMA), the cTBS-cM1 group (cTBS targeting contralesional M1), and the cTBS-cPMA group (cTBS targeting contralesional PMA). All subjects will undergo comprehensive rehabilitation and the corresponding cTBS interventions once a day, five times a week for 4 weeks. Clinical scales, neurophysiological examinations, and neuroimaging will be used as evaluation tools in this study. As the primary outcome, clinical performance on muscle spasticity of elbow/wrist flexor/extensors and upper-limb motor function will be evaluated with the modified Ashworth scale and the Fugl-Meyer Assessment of Upper Extremity Scale, respectively. These scale scores will be collected at baseline, after 4 weeks of treatment, and at follow-up. The secondary outcomes were neurophysiological examinations and Neuroimaging. In neurophysiological examinations, motor evoked potentials, startle reflex, and H reflexes will be used to assess the excitability of the subject's motor cortex, reticulospinal pathway, and spinal motor neurons, respectively. Results of them will be recorded before and after the first cTBS treatment, at post-intervention (at 4 weeks), and at follow-up (at 8 weeks). Neuroimaging tests with diffusion tensor imaging for all participants will be evaluated at baseline and after the 4-week treatment.

**Discussion:**

Based on the latest research progress on post-stroke spasticity, we innovatively propose a new neuromodulation target for improving post-stroke spasticity *via* cTBS. We expected that cTBS targeting cPMA would have significant immediate and short-term effects on spasticity and related neural pathways. The effect of cTBS-cPMA may be better than that of cTBS *via* conventional cM1. The results of our study will provide robust support for the application of cTBS neuromodulation in post-stroke spasticity after a severe stroke.

**Clinical trial registration:**

This trial was registered with chictr.org.cn on June 13, 2022 (protocol version). http://www.chictr.org.cn/showproj.aspx?proj=171759.

## Introduction

Post-stroke spasticity is defined as a velocity-dependent increase in exaggerated stretch reflex responses due to the hyperexcitable descending excitatory brainstem pathways and results in the hyperexcitability of alpha motor neurons in the spinal cord (1). Spasticity is a common complication after stroke, with the prevalence ranging from 30% to 80% of stroke survivors (2). The appearance of spasticity after stroke can significantly affect the patient's recovery of motor function and cause long-term problems such as joint contractures, abnormal pain, and deformity (3). The spasticity of most patients with mild to moderate stroke will gradually improve during the movement recovery process, but that of some patients with severe injury will progress in the opposite way (4). Reorganization of the descending cortical spinal tract (CST) and reticular spinal tract (RST) from both hemispheres may be a plausible explanation for this distinct development (5).

Both CST and RST are the two important descending pathways of postural and motor control in primates. RST is usually considered to control proximal and axial muscles, and to be involved mainly in movement and posture adjustment, while CST is thought to be involved in fine motor control (6). The two tracts are competitive but in a wonderful state of balance. Significant brain lesion after stroke causes structural damage to these descending pathways, especially in the compact pyramidal cell populations such as CST (7). However, the RST dominated by ipsilateral innervation has relatively small damage, and its participation in motor control is relatively increased and manifested in the patient's movement, such as the clumsy and synergic movement of the upper limbs (8, 9). Spasticity after a stroke occurs and diminishes in synchrony with the appearance and disappearance of these pathological movement patterns (10, 11). Although it is still not clear how the regulation of muscle tone by the two major systems is carried out, it is speculated that the loss of inhibitory regulation caused by the imbalance of CST and RST triggers the abnormal hyperactivation of the medial reticulospinal tract (mRST) (9, 11–13). This imbalance is especially pronounced in patients with severe brain lesions. Recent imaging evidence suggests that ipsilateral PMA is the advanced cortex of RST in motor control, which plays a key role in ipsilateral movement (14, 15). After cortical damage, the contralesional PMA area is hyperactivated and may trigger a weakened inhibition of the ipsilateral mRST pathway (12). Due to the persistence of severe injury, the injured CST is difficult to recover, and this persistent weakening of inhibition can be inappropriately reinforced during the neural reorganization of the contralesional RST and related cortices (1). With appropriate treatment, attenuating the hyperexcitability of this pathway *via* contralesional PMA may be a therapeutic idea to stop spasticity from exacerbating.

Limited interventions were proven effective currently in improving spasticity. The only botulinum toxin injection backed by a lot of evidence is expensive and may require multiple iterations (16). Some central modulating strategies have also been attempted to treat post-stroke spasticity. Transcranial magnetic stimulation (TMS) is one of the options. However, ambiguity in research results leads to the disapproval of this technology on post-stroke spasticity (17, 18). The reason for this may be that previous study designs did not take into account the dynamic reorganization of CST/RST, and mainly focused on the promotion of motor recovery (1). Almost all studies with positive results were interventions targeting the bilateral M1 areas (part of CST) (19–21), improvement in spasticity accompanied by significant motor recovery. This can be interpreted as a weakening of RST regulation by enhancing CST control, which may not the most direct way to regulate spasticity-related pathways. Moreover, the traditional inhibitory TMS therapy developed based on the theory of hemispheric inhibition (22) was insufficient to reverse cortical excitability in all subjects or produced short-lived or variable results (23). Continuous theta burst magnetic stimulation (cTBS) is a novel patterned protocol of TMS, it can induce long-term depression (LTD) effect on cortical excitability and lead to long-term therapeutic benefits in facilitating induction of neuronal plasticity mechanisms (24). TBS has the advantages of shorter stimulation duration, low stimulation pulse intensity, longer post-acting time, and probably improved efficiency compared with ordinary rTMS, which is regarded as a very promising therapy (25). In addition, how to assess post-stroke spasticity objectively also set a barrier for related research. Most previous studies of TBS on post-stroke spasticity mainly use the modified Ashworth scale (MAS) as their primary outcome (18), which only roughly reflects changes in spasticity, and does not benefit mechanistic exploration.

Combined with the recent mechanism research on post-stroke spasticity, we propose a novel central regulatory target for cTBS treatment of post-stroke spasticity, namely cPMA. At the same time, the contralateral M1 area, which was extensively used in previous studies, was also introduced into our study design. Based on a sham control, we will explore the effects of “direct” intervention (cTBS *via* cPMA) and “indirect” intervention (cTBS *via* cM1) on post-stroke spasticity and verify our hypothesis on the abnormal neural regulation of spasticity after stroke.

In addition to clinical assessment scales, we will introduce neurophysiological examinations and diffusion tensor imaging (DTI) for this study. Motor evoked potentials (MEP), startle reflexes (SR), and H reflex will be used to assess the excitability of the subject's motor cortex, reticulospinal pathway, and spinal motor neurons, respectively. DTI will be performed to show the contralesional neural reorganization.

We hypothesize that cTBS in cPMA ameliorates spasticity in patients with severe subacute stroke by inhibiting the overactivated mRST and reducing the excitability of spinal motor neurons, thereby improving motor outcomes of the upper extremity. This study aims to evaluate the efficacy of cTBS therapy in the contralesional PMA for severe post-stroke spasticity and confirm the above hypothesis.

## Methods and analysis

### Study design

**Figure 1:**
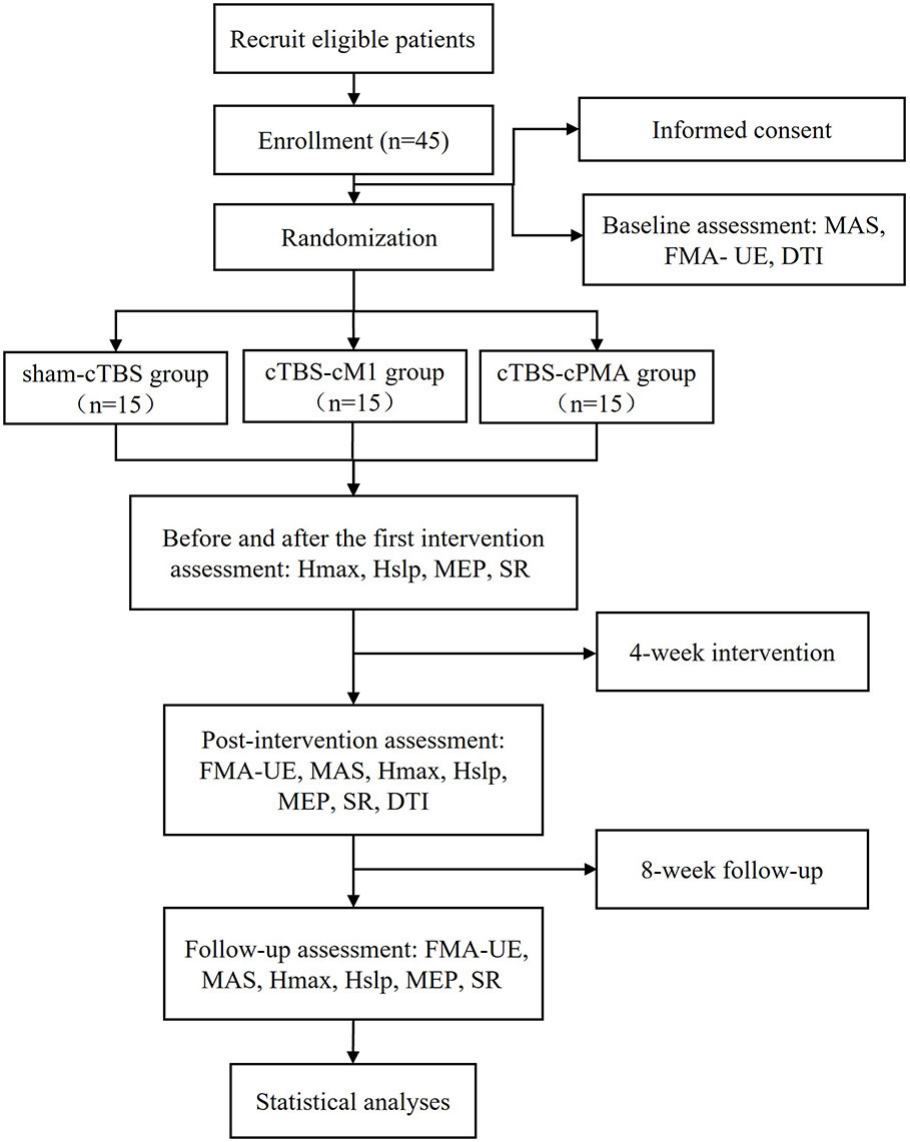
The flow diagram for this study. MEP, motor evoked potential; MAS, modified Ashworth scale; FMA-UE, Fugl-Meyer Assessment of Upper Extremity Scale; SR, startle reflex; DTI, diffusion tensor imaging; Hslp/Mslp, the ratio of the slope of H and M-waves; Hmax/Mmax, the ratio of the maximum of H and M-waves.

The study will be a non-blinded pilot randomized controlled trial with three parallel groups. The study will be performed in accordance with the Declaration of Helsinki. This protocol follows the Consolidated Standards of Reporting Trials (CONSORT) Statement on randomized trials and it will be conducted according to the Recommendations for Interventional Trials (SPIRIT). The trial was registered in the Chinese Clinical Trial Registry (No. 2200060885). It will be carried out in the Department of Rehabilitation Medicine of Tongji Hospital in Wuhan, China. A flowchart overview of the study is presented in Figure 1. The Standard Protocol Items: SPIRIT table for enrolment, interventions, and assessments is presented in Table 1. Forty-five eligible subjects will be randomly allocated into three groups equally: (1) sham-cTBS group, (2) cTBS-cM1 group (cTBS targeting contralesional M1), (3) cTBS-cPMA group (cTBS targeting contralesional PMA).

**Table 1:** Tabulation of enrolment, interventions, and assessments throughout the trial.

	**Study period**					
	**Enrolment**	**Allocation**	**Before and after the first intervention**	**Intervention**	**Post-intervention assessment**	**Follow-up**
Timepoint	−1 week	0	1 day	0–4 week	4 week	8 week
**Enrolment**						
Eligibility screen	**×**					
Informed consent	**×**					
Allocation		**×**				
**Interventions**						
sham-cTBS group				**×**		
cTBS-cM1 group				**×**		
cTBS-cPMA group				**×**		
**Assessments**						
MAS		**×**			**×**	**×**
FMA-UE		**×**			**×**	**×**
MEP			**×**		**×**	**×**
SR			**×**		**×**	**×**
Hmax/Mmax			**×**		**×**	**×**
Hslp/Mslp			**×**		**×**	**×**
DTI		**×**			**×**	

Tabulation of enrolment, interventions, and assessments throughout the trial. cTBS, continuous theta burst stimulation; MEP, motor evoked potential; MAS, modified Ashworth scale; FMA-UE, Fugl-Meyer Assessment of Upper Extremity Scale; SR, startle reflex; DTI, diffusion tensor imaging; Hslp/Mslp, the ratio of the slope of H and M-waves; Hmax/Mmax, the ratio of the maximum of H and M-waves.

### Participants

The inclusion criteria are as follows: (1) age 18–80 years old. (2) first unilateral stroke (cerebral infarction, primary intracerebral hemorrhage, subarachnoid hemorrhage) confirmed by MRI or CT within 6 months before enrolment. (3) Perceptible muscle spasticity with MAS score >1. (4) Severe motor impairment with the Fugl-Meyer Assessment of Upper Extremity (FMA-UE) score 0–35 (9, 26). (5) Failure to induce MEP in the upper extremity of the hemiplegic side (27, 28). (6) Mini-Mental State Examination (MMSE) score ≥ 22 and compliance with the interventions. (7) sign the informed consent.

The exclusion criteria are as follows: (1) more than one stroke (patients with previous transient ischemic attack could participate), bilateral cerebral hemisphere lesion. (2) history of seizures or seizures after stroke, severe skull fracture. (3) conditions unsuitable for TBS (29), e.g., intracranial implants, cardiac pacemakers or pregnancy, implanted drug pumps, etc. (4) Medications (such as lignocaine, penicillins, cephalosporins, amphotericin, tricyclics, selective serotonin reuptake inhibitors, azatadine, aminophylline, bupropion, imipramine, clozapine, olanzapine, etc.) that may lower the seizure threshold have been used continuously for nearly 3 months. (5) serious impairments of heart, lung, liver, kidney, and other organs, cannot tolerate training. (6) participation in other spasticity rehabilitation studies.

### Randomization and blinding

The physician will screen out the patients who meet the eligibility criteria, explain the whole content of the trial and ask the consent to sign the informed consent. Their demographic information, medical history, and medication details will then be collected. We will use an electronic random sequence generator (www.random.org) to randomly assign all of the individuals to one of the three groups in a 1:1:1 ratio. An independent researcher will conceal the allocation using sealed and opaque envelopes, numbered consecutively. In this trial, subjects, assessors, and data analysts are blind to the allocation results. Because this study involves locating therapeutic targets in the cerebral cortex, the intervenor cannot be blind. The treatment and outcome evaluation regimen are depicted in Table 1.

### Procedure

The study will consist of four phases: (1) A baseline assessment involving clinical scales (MAS and FMA-UE) and DTI before the stimulation sessions; (2) Comprehensive rehabilitation, and the corresponding cTBS interventions will be provided once a day, 5 times a week for 4 weeks. Neurophysiological assessments such as H reflex, MEP and SR will be collected before and after the first cTBS treatment at this phase; (3) Post-intervention (4 weeks) clinical, neurophysiological and DTI assessment; (4) Follow-up (8 weeks) clinical and neurophysiological assessment. Figure 1 shows the flow diagram of this study.

All stroke patients will receive a routine treatment programme including rehabilitation, medication, multidisciplinary therapy. The patient's rehabilitation included various forms of physical and occupational therapy, ~3 to 4 hours a day, 5 days a week, for 4 weeks. The intensity of active training will be appropriately adjusted according to the patient's tolerance. At the same time, an additional cTBS treatment will be provided for those participants for 4 weeks, once a day. Each patient's cTBS intervention will be performed by the same therapist.

**Figure 2:**
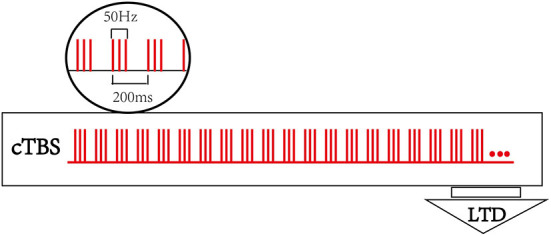
Continuous theta burst stimulation (cTBS) paradigm and neurophysiological effects. The basic element of cTBS is a burst of 3 stimuli at 50 Hz, which is repeated every 200 ms. In cTBS paradigm, an uninterrupted 40 s train of TBS was delivered (600 pulses).

A TMS-navigation system (Localite TMS navigator, Germany) will be used to locate the therapeutic targets, record data, and delivery the stimulation. Each patient's MRI data will be collected and imported into the navigation system to build a personalized brain model. Participants will be asked to sit in a comfortable chair with adhesive surface electrodes placed over the muscle belly and tendon of the first dorsal interosseous (FDI). The cortical target eliciting the largest responses and minimum latency of the motor evoked potential (MEP) in the first dorsal interosseous (FDI) muscle at resting status will be determined as the hotspot. The resting motor threshold (rMT) of contralesional hemisphere will be recorded accordingly, which is defined as the lowest stimulus intensity producing a MEP (peak to peak amplitude ≥ 50 μV in at least 5 of 10 trials) in the relaxed condition (30). T.M.S Motor Threshold Assessment Tool (MTAT) 2.0 (http://www.clinicalresearcher.org/software.html) will be used to assess the motor threshold (MT) (31). The location of the FDI hotspot will be set as the cTBS target for cTBS-cM1 group. As to the treatment target for cTBS-cPMA group, the dorsal portion of the superior precentral sulcus (above the superior frontal sulcus) in the contralesional hemisphere will be selected as the landmark for targeting the PMA (32). The sham group will receive cTBS with the same parameters (intensity, time) as the cTBS group on PMA of the unaffected hemisphere but with the coil rotated 90° away from the scalp so that the induced current is minimized. A transcranial magnetic stimulation (TMS MagVenture^®^ MagPro R30, Denmark) with a cool-B65 A/P figure-of-eight-shaped coil will be applied as the stimulator, and the stimulus intensity is set to 70% of contralesional hemisphere's rMT for the three groups. The participants will receive an uninterrupted 40 s train of sham cTBS stimulation over contralesional PMA or an uninterrupted 40 s train of cTBS over contralesional M1 or PMA, respectively. The basic element is a burst ofthree stimuli at 50 Hz, which is repeated at intervals of 200 ms (i.e., 5 Hz) (24), for a total of 600 pulses. Figure 2 shows the cTBS paradigm.

### Tolerability and safety

Participants will be monitored during the treatment to identify any signals or adverse reactions, such as pain on stimulation site, headache, nausea, and very unlikely seizures (0.08/1,000 sessions) (33), that would require their exclusion from the study. Furthermore, any discomfort experienced by the participants will be recorded and reported.

### Outcome measurements

The primary outcomes are MAS and FMA-UE. Changes before and after the 4 weeks of treatment will be used as the primary measure to assess improvement in spasticity and abnormal movement synergies (9). And the secondary outcomes are results from neurophysiological examinations including H reflex, M response, MEP and SR, and changes of neuroimaging with DTI.

#### Clinical assessments

##### Modified Ashworth Scale

The Modified Ashworth Scale (MAS) is widely used to evaluate muscle spasticity by measuring resistance during passive soft-tissue stretching. The MAS has a six-point scale (ranging from 0 to 4, respectively 0, 1, 1^+^, 2, 3, 4) with a score of 0 representing no spasticity and a score of 4 representing severe spasticity (34, 35). An experienced clinician will use MAS to evaluate the muscle tone of the elbow flexor, elbow extensor, and wrist flexor and extensor on the hemiplegic side of the patient and record them, respectively.

##### Fugl-Meyer Assessment of Upper Extremity Scale

We will also assess the mean change in the Fugl-Meyer Assessment of the Upper Extremity (FMA-UE) Scale. This is an assessment table for the upper extremity, upper arm, and wrist/hand, which examines motion, coordination, and reflexes. The FMA-UE scale has three points (respectively, 0, 1, 2 score, 0 is the worst, 2 is the best) for each item with a maximum of 66 points. It is widely used to measure post-stroke motor recovery of the upper limb because of its convenience, effectiveness, and reliability, and the higher the score, the greater the motor function (36, 37). Some of the recent refinement of flexor or extensor synergy movements and out-of-synergy movements based on the movements assessed in FMA-UE will help reflect changes in motor innervation pathways through score changes (9).

Those clinical assessments will be performed at baseline, after 4 weeks of treatment, and at follow-up.

#### Neurophysiological examinations

##### CST excitability: Motor evoked potential

TMS evoked neurophysiological parameters are useful measures for monitoring post-stroke patients and predicting recovery of motor function (38). MEP is used to investigate CST activities frequently. In this study, we will collect the contralesional hemisphere's resting motor threshold (rMT) and motor evoked potentials (MEP) from the first dorsal interosseous (FDI) muscles. All subjects will be seated in a comfortable chair with their hands completely relaxed during the evaluations. The transcranial magnetic stimulation coil will be held tangentially in a posterior anterior plane at a 45° angle from the midline over the hotspot. As mentioned before, we will primarily find the hot spots of FDI, and measure rMT. After that, MEPs will be sampled during TMS at 110% rMT and will be kept constant during the following evaluations. We will record the MEP as zero if it cannot be evoked (39).

##### RST excitability: Startle reflex

The subject will be instructed to sit in a chair with a backrest, with the hands resting on the thighs and remaining relaxed. Two pairs of surface electromyography electrodes were applied to the left and right sternocleidomastoid muscles (SEM) belly record muscle response. After wearing a headphone (Sennheiser HD25-I; Wedemark, Germany), the subjects will receive a total of 30 sound stimuli. Half of them are 40 ms beeps at 80 dB and half are 40 ms white noises at 120 dB. The stimulus firing sequence was randomly generated, with a random interval of 6–10 s between stimuli to ensure subjects were fully relaxed. The Psychtoolbox-3 toolkit within MATLAB (2017b, MathWorks, Natick, MA, USA) will be used for the design and execution of this trial. The sEMG signals will be collected by the Ultimu EMG system (Noraxon USA Inc., Scottsdale, AZ, USA) at a sampling rate of 2,000 Hz. Raw sEMG data will be output and processed under the same software. It will be segmented, bandpass filtered at 30–300 Hz, and notch filtered at 50 Hz. A threshold detection method (mean with 3SD) based on the amplitudes at time window 2,500–500 ms before each stimulus will be set to determine the onset of muscles (40). A positive startle response will be screened out if the onset of either SEM at the time widow of 30–130 ms after stimuli. The positive rate, as well as SEM onset delays (41) will be recorded for analysis.

##### Motor neuron pool excitability: H reflex and M response

Hslp/Mslp (the ratio of the slope of H and M-waves) denotes the relationship between stimulus intensity and motor neuron reflex recruitment (42), and previous researches have proposed Hslp/Mslp and Hmax/Mmax (the ratio of the maximum of H and M-waves) as sensitive and objective measures for the motor neuron pool excitability (42, 43). Poststroke patients have poor autonomic activity, which is thought to contribute to increased reflex excitability manifested by greater Hslp and Hmax on the paretic side (44–46).

A physician will examine all patients with Dantec, two channel EMG. All subjects will be seated comfortably in a chair with their hands resting on thighs and elbows flexed 20°-30°. Paired surface electrodes will be attached to the skin on the belly of the flexor carpi radialis (FCR) muscle. We will stimulate (1 ms duration, once every 3 s) the median nerve at medial to the bicep tendon in the antecubital fossa to elicit H reflexes and M-responses from the flexor carpi radialis muscle. The intensity of these pulses will increase gradually from below the threshold of the H-reflex to supramaximal and saturated condition for M response. At each stimulus intensity, we will deliver 3–5 pulses successively. The H-reflexes and the M responses evoked by these pulses will be amplified in the bandwidth 5 Hz−3 kHz. All amplification procedures are controlled by software running on a signal processor. All signals will be checked and derived to a hard disk for offline analyses. All data regarding the size and series stimulus intensities of both the H-reflex and M response will be used in the construction of the respective recruitment curves and to calculate the average of their maximum values.

Electrophysiological examinations will be performed before and after the first cTBS treatment, at post-intervention (at 4 weeks), and at follow-up (at 8 weeks).

#### Neuroimaging: diffusion tensor imaging

Diffusion Tensor Imaging (DTI) will be evaluated at baseline and after the 4-week treatment. And it will be conducted as follows. All individuals will be subjected to 3.0 T MRI scanner (Discovery LS MR 750; GE Healthcare, Chicago, IL, USA) equipped with a 16-channel head & neck coil to acquire 3D T1-weighted and DTI images before and after 20 times therapy.

DTI is used in diffusion-weighted gradients along 60 non-collinear directions and two non-diffusion-weighted volumes. Scanning parameters will include matrix size = 128 × 128, field of view = 230 × 230 mm^2^, TR = 5,800 ms, TE = 71 ms, flip angle = 180°, number of averages = 1, b value = 1,000 s/mm^2^, slice thickness = 4 mm, and voxel size = 1.7 × 1.7 × 4 mm^3^ (47).

DTI data processing will be carried out as follows. (1) Normalization: All data will be spatially normalized using parameters derived from 3D T1WI processing. We will use FMRIB's Diffusion Toolbox and TBSS (Tract-Based Spatial Statistics) (both from the FMRIB Software Library 6.0, FSL, https://fsl.fmrib.ox.ac.uk/fsl/fslwiki/FSL) to analyze DTI data. (2) Registration: We will use FSL tools to get the transformation matrix and then correct the eddy current distortions in the DTI datasets. After that, each subject's raw diffusion-weighted pictures will be linearly aligned to a non-diffusion weighted image (b0) with the removal of the non-brain tissues using a brain extraction tool (BET). (3) Local fitting: The extracted brain will be used for diffusion tensors. The diffusion tensor will be estimated at each voxel to obtain the fractional anisotropy (FA) pictures (47). We will select the region of interest (ROI) method to measure tract integrity. The red nucleus (RN) is an appropriate part to draw ROI (7). We will draw ROI on the posterior limb of internal capsule and the anterior pons on a color-coded map for CST and on the RN for RST individually. In each ROI, we will extract fractional anisotropy (FA), mean diffusivity (MD), radial diffusivity (RD) and axial diffusivity (AD). Three dimensional reconstructions of the CST and RST will be performed for each patient.

DTI has been commonly used to quantitatively measure the integrity of tissues. Previous studies reported the DTI biomarkers (e.g., FA) measured after stroke have emerged as potential predictors of motor recovery (48, 49).

### Sample size calculation

Sample size is estimated through software G^*^Power 3.1 (50). We used MAS as the primary outcome for the calculation of the effect size. It is determined to be 12 per group based on a power of 0.8 with α level at 0.05. According to a previous study (21), effect size *d* = 0.53 was calculated based on the difference in MAS score from their main research results. Through conversion, the effect size *f* used in our study is about 0.25. Repeated measures correlation in our study is set at 0.5 to detect a moderate within-between effect of Conhen's *f* = 0.25. Considering a drop-out rate of 20%, we aim to recruit totally 45 stroke subjects.

### Data monitoring and management

The data monitoring committee (DMC) 's responsibilities include safety monitoring, test data monitoring and test design adjustment recommendations with no conflicts of interest. Rehabilitation Department of Tongji Hospital will be in charge of informed consent quality assurance, eligible participant recruiting, implementation of interventions, and data administration. Designated person will be responsible for collecting case report forms (CRFs) as well as data transfer and analysis. The investigator and director are responsible for retaining all records, and the data center will keep anonymized case report form data. Electronic data will be preserved on password-protected computers, while all paper data will be kept in secure filing cabinets.

### Statistical analysis

The SPSS 21.0 program will be used for all statistical analysis and the significance level set for all the analysis will be *P* ≤ 0.05. The Shapiro-Wilk test will be used (*P* > 0.05) to check normality. In this study, Continuous variables will be expressed as mean with standard deviation and median for non-normal distributions and categorical variables will be presented as percentage or frequencies. Between the three groups, data will be first compared using Student's *t*-test for continuous variables and the chi-square or Fisher's exact tests for categorical variables. Appropriate metrics will be processed further using repeated measures ANOVA. Bonferroni correction method and False Discovery Rate <5% will be considered for multiple comparisons. All analyses will be done with the intention to treat principle, and missing data will be estimated using multiple imputation. The mean comparisons for related outcomes in two different moments will be studied with the Wilcoxon-signed rank test. The major strategy for dealing with missing data will be a sensitivity analysis and weighted estimating equations.

## Discussion

The motor recovery after stroke follows a basically predictable pattern, from flaccid, spastic to recovered (4, 10). In patients with severe stroke, motor function may be arrested at a certain stage limited by spasticity for a long time in the process of motor recovery (4), which is a thorny problem in the rehabilitation process. Motor rehabilitation relies on a complex combination of spontaneous recovery and motor learning to promote neural plasticity (11, 51). The spinal plastic change relates to the severity of cortical damage and has a time-limited window, almost entirely in the first weeks (52, 53). The anatomical structure determines that in patients with severe stroke, CST is more vulnerable to damage, ultimately leading to the facilitatory medial RST being unopposed and independent of cortical control (13), which plays a significant role in spasticity. Spasticity reflects a phenomenon of maladaptive neuroplasticity in motor control pathways (12), and can easily develop if there is not appropriate intervention to regulate in the course of recovery (54).

Non-invasive magnetic stimulation is a promising therapy option for modulating neural plasticity, but the undetected MEP (27) and the extensive recombination of the cerebral cortex (55) after severe stroke lead to doubtful targeting in the cortex for treatment. Although there is weak-grade evidence to support that the contralesional TMS targeting cM1 improves post-stroke spasticity, the effect is inconclusive (54). Exploring the spasticity mechanism in severe stroke patients may be more helpful to obtain optimized intervention targets and clarify the effect of this non-invasive treatment.

As mentioned previously, spasticity is thought to be a top-down manifestation resulting from hyperexcitability of the advance central PMA and its corresponding descending pathway of medial RST (12). This study will compare the acute and short-term effects of cTBS over cPMA and M1 in severe stroke patients. Moreover, we aim to demonstrate that the hyperexcitable cPMA after stroke is the cortical manifestation causing spasticity, and reducing the hyperexcitability of cPMA may help to down regulating the excitability mRST of hemiplegic side and to facilitate adaptive neuroplasticity of RST in spasticity relief and motor recovery.

Several characteristics distinguish this study from previous reports. Firstly, it is the first clinical study that TBS improves spasticity by regulating the neuroplasticity of cPMA and mRST. The results will verify the relationship between spasticity, cPMA and mRST, and provide theoretical and practical support for effective improvement of spasticity. Otherwise, the changes of neuroplasticity and spasticity after stroke will be evaluated objectively and comprehensively from clinical, electrophysiological and neuroimaging perspectives. In this study, we use the FMA-UE scale to monitor the recovery course of the upper extremity's motor function (56). Moreover, subsections and items of FMA-UE such as synergy or out-of-synergy movements can roughly reveal the pathways (CST/RST) that patients currently dominate their motor and spastic recovery (9). Some personalized rehabilitation decisions based on this feature may help develop more effective treatment plans. DTI biomarkers of CST have been considered as predictors of motor recovery (48). In this study, DTI will be used to evaluate the neuroplasticity changes of both RST (47) and CST (48) in post-stroke spasticity. The results will be used to further clarify the dynamic competitive relationship between the both (9). We expect that the results of this study can provide powerful support for the application of TBS neuromodulation in improving spasticity, and we may supplement targeted training [motor training, auditory stimulation training (57), etc.] in different stages of motor recovery in the near future, so that stroke patients can reasonably utilize CST and RST pathways to facilitate motor function recovery.

## Ethics statement

The studies involving human participants were reviewed and approved by Institutional Ethical Committee of Tongji Hospital. The patients/participants provided their written informed consent to participate in this study.

## Author contributions

YL, WG, NX, and XW contributed to the conception and design of the study. XW and NX wrote the first draft of the manuscript and generated the figures. All authors contributed to the refinement of the study protocol and approved the final manuscript.

## Conflict of interest

The authors declare that the research was conducted in the absence of any commercial or financial relationships that could be construed as a potential conflict of interest.

## Publisher's note

All claims expressed in this article are solely those of the authors and do not necessarily represent those of their affiliated organizations, or those of the publisher, the editors and the reviewers. Any product that may be evaluated in this article, or claim that may be made by its manufacturer, is not guaranteed or endorsed by the publisher.

## Abbreviations

Abbreviations: CST: corticospinal tract
RST: reticulospinal tract
cPMA: contralesional premotor area
cM1: contralesional primary motor cortex
mRST: the medial reticulospinal tract
LTD: long-term depression
FMA-UE: Fugl-Meyer Assessment of Upper Extremity
MEP: motor evoked potential
SR: startle reflex
MAS: Modified Ashworth scale
DTI: Diffusion tensor imaging
Hslp/Mslp: the ratio of the slope of H and M-waves
Hmax/Mmax: the ratio of the maximum of H and M-waves
RN: red nucleus.
